# Chemical Composition Antioxidant and Anti-Inflammatory Activities of *Myrtus communis* L. Leaf Extract: Forecasting ADMET Profiling and Anti-Inflammatory Targets Using Molecular Docking Tools

**DOI:** 10.3390/molecules29040849

**Published:** 2024-02-14

**Authors:** Samia Belahcene, Widad Kebsa, Tomilola Victor Akingbade, Haruna Isiyaku Umar, Damilola Alex Omoboyowa, Abdulaziz A. Alshihri, Adel Abo Mansour, Abdulaziz Hassan Alhasaniah, Mohammed A. Oraig, Youssef Bakkour, Essaid Leghouchi

**Affiliations:** 1 Laboratory of Biotechnology, Environment and Health, Faculty of Nature and Life Sciences, University of Jijel, Jijel 18000, Algeria; 2 Laboratory of Molecular Toxicology, Faculty of Nature and Life Sciences, University of Jijel, Jijel 18000, Algeria; w.kebsa@univ-jijel.dz; 3 Computer-Aided Therapeutic Discovery and Design Platform, Federal University of Technology, PMB 704 Akure, Gaga 340110, Nigeria; victomilola@gmail.com (T.V.A.); uhumar@futa.edu.ng (H.I.U.); 4 Phyto-Medicine and Computational Biology Laboratory, Department of Biochemistry, Adekunle Ajasin University, Akungba Akoko 57257, Nigeria; 5 Department of Radiological Sciences, College of Applied Medical Science, King Khalid University, Abha 61421, Saudi Arabia; aaalshehre@kku.edu.sa; 6 Department of Clinical Laboratory Sciences, College of Applied Sciences, King Khalid University, Abha 61421, Saudi Arabia; 7 Department of Clinical Laboratory Sciences, College of Applied Sciences, Najran University, Najran 1988, Saudi Arabia; 8 Radiology Department, Khamis Mushayt General Hospital, Khamis Mushayt 62433, Saudi Arabia; moraig@moh.gov.sa

**Keywords:** *Myrtus communis* L., methanolic extract, bioactive molecules, antioxidant, anti-inflammatory, molecular docking, ADMET

## Abstract

Compounds derived from natural sources continue to serve as chemical scaffolds for designing prophylactic/therapeutic options for human healthcare. In this study, we aimed to systematically unravel the chemical profile and antioxidant and anti-inflammatory activities of myrtle methanolic extract (MMEx) using in vitro, in vivo, and in silico approaches. High levels of TPC (415.85 ± 15.52 mg GAE/g) and TFC (285.80 ± 1.64 mg QE/g) were observed. Mass spectrophotometry (GC-MS) analysis revealed the presence of 1,8-cineole (33.80%), α-pinene (10.06%), linalool (4.83%), p-dimethylaminobenzophenone (4.21%), thunbergol (4%), terpineol (3.60%), cis-geranyl acetate (3.25%), and totarol (3.30%) as major compounds. MMEx induced pronounced dose-dependent inhibition in all assays, and the best antioxidant activity was found with H_2_O_2_, with an IC_50_ of 17.81 ± 3.67 µg.mL^−1^. MMEx showed a good anti-inflammatory effect in vivo by limiting the development of carrageenan-induced paw edema. The pharmacokinetic profiles of the active molecules were determined using the SwissADME website, followed by virtual screening against anti-inflammatory targets including phospholipase A2 (PLA-2), cyclooxygenase-2 (COX-2), tumor necrosis factor alpha (TNF-α), interleukin-1β (IL-1β), and NF-κB. A pharmacokinetic study revealed that the molecules have good absorption, distribution, and metabolism profiles, with negative organ toxicity. Among the compounds identified by GC-MS analysis, pinostrobin chalcone, cinnamyl cinnamate, hedycaryol, totarol, and p-dimethylaminobenzophenone were observed to have good binding scores, thus appreciable anti-inflammatory potential. Our study reveals that MMEx from Algerian *Myrtus communis* L. can be considered to be a promising candidate for alleviating many health complaints associated with oxidative stress and inflammation.

## 1. Introduction

Even though free radicals can operate as redox-signaling messengers, oxidative stress can also orchestrate the complex pathophysiological mechanisms in many diseases [1]. An imbalance between pro-oxidant and antioxidant molecules affects the redox circuitry and cell integrity through a toxic onslaught on non-target tissues [2]. A stochastic accumulation of reactive oxygen species (ROS) and their precursors stimulates the expression of redox-sensitive pro-inflammatory cytokines and caspases [3]. Oxidative stress and inflammation are interrelated in an ambivalent way, since one can promote the other, leading to a “toxic feedback” system [4]. An acute inflammatory cascade is a protective process characterized by a spatiotemporal orchestration of enzymatic reactions and cellular activation [5]. It starts abruptly and generally resolves quickly [6], resulting in tissues being returned to functional homeostasis with an endogenous programmed resolution [7,8]. It progresses through the generation of a large pool of arachidonic acid (AA) from membrane phospholipids by the action of phospholipase A2 (PLA2) [9]. Subsequently, AA is metabolized by cyclooxygenase (COX), resulting in the production of eicosanoids such as leukotrienes (LTs), prostaglandins (PGs), and thromboxane (TX) [10]. The primary inflammatory stimuli, including interleukin-1β (IL-1β) and tumor necrosis factor alpha (TNF-α), which are cell-signaling proteins (cytokines) responsible for the acute phase reaction by activating typical NF-κB signaling and related receptors, are closely linked to oxidative stress mechanisms [11]. If acute inflammation is not resolved, it becomes destructive, more complex, and sophisticated [12]. This scenario is likely to amplify drastically due to the prevalence of uncontrollable increased cytokine release (cytokine storm), leading to long-lasting inflammation [13]. Chronic inflammation also produces free radicals, which ultimately aggravate the inflammatory status [14].

In the pharmaceutical industry, archetypal non-steroidal anti-inflammatory drugs (NSAIDs) have been extremely popular for their certified ability to prevent inflammation; they do this by extinguishing COXs, which in turn inhibit the formation of PGs and PLA2 to suppress the production of other pro-inflammatory mediators [15,16]. The unrestrained increase in the use of NSAIDs over the years is still controversial, since there are an escalating incidences of toxicity [17]. Unexpectedly, adverse gastrointestinal reactions, including occult bleeding, so-called enteropathy [18], kidney failure [19], and liver damage [20], were reported to be the most pervasive and unavoidable side effects of the prolonged consumption of NSAIDs, putting people all over the world in jeopardy. Notably, the alarming spread of adverse toxicity in certain segments of the population, the high cost, and the upsurge in drug resistance are three specific hindrances to increasing the use of NSAIDs, which are becoming less supportive with a narrow safety window. With an increased collective consciousness among people, a belief has taken root that everything “natural” is undoubtedly healthy [21]. Against the backdrop of these contrasting circumstances, a number of research teams are eagerly searching for a prophylactic therapeutic strategy from nature that has a significant safety margin when applied to both experimental animals and humans [22,23].

*Myrtus communis* L. (family: Myrtaceae) is a useful ethnomedicinal shrub explicitly grown in districts in Algeria that are native to Mediterranean ecosystems, with a broad ecological amplitude adapted to various climatic conditions [24]. Historically, it was widely used as medicine under the vernacular name “El-Rayhan”. Areal parts of myrtle have been used as a decoction, an infusion, and a health remedy for bathing newborns with inflamed skin and washing sores [25,26]. Additionally, it was used to treat oral wounds, disorders of the digestive and urinary systems [25], diarrhea, peptic ulcers, hemorrhoids [27], and inflammations [28,29]. Due to its qualified safety, there have been intensified efforts to determine the chemical profile and various biological effects of myrtle extracts. Hence, the ethnopharmacological value of this plant derives from its ubiquitously abundant phytochemicals, including poly(phenols), flavonoids, tannins, gallic acid, flavonol derivatives, hydroxybenzoic acids alkaloids, terpenoids, and quinonoids [30]. Under rigorous scientific scrutiny, promising antibacterial [31], anti-inflammatory [28,32], anticancer [33], antioxidant, antidiabetic [24], antigenotoxic [34], and antimicrobial activities [35] of *M. communis* were discovered with multi-component, multi-target, and multi-mechanism models, making it an ideal choice to be exploited for the development of novel therapies [36]. Myrtle has been deeply scrutinized by the research community, whereas in Algeria, the chemical profile and biological activities of myrtle leaves have not been explicitly investigated. There is no plausible evidence explaining its anti-inflammatory behavior at the molecular level. This study aims to elucidate these mechanisms in order to bridge the research gap and provide deep insights into how MMEx can be harnessed for specific therapeutic applications.

The exceptional advancements in computational approaches represent an opportunity to identify and design pharmacologically active natural molecules that can target proteins of interest [37]. The use of in silico tools has recently opened new avenues for research in the realm of pharmacotoxicology. Consequently, there is currently an increased focus on developing effective, safe, and low-cost therapeutic modalities with the help of cheminformatics tools, including pharmacokinetic and molecular docking, in order to provide valuable insights into complex and experimentally difficult phenomena such as enzyme reaction mechanics and ligand–receptor linkages [6,38].

The objective of this study is to provide a holistic and comprehensive view of the pharmacological potential of MMEx, first by deciphering and identifying the phytochemical composition, and secondly by evaluating the in vitro, in vivo, and in silico antioxidant and anti-inflammatory activities involved. Furthermore, the ADMET profiling and drug-likeness of the identified phytochemicals are rationalized using molecular docking tools.

## 2. Results

### 2.1. Extraction Yeild (EY) and Total Polyphenol, Flavonoid, and Tannin Contents

The extraction yield of *Myrtus communis* L. methanolic extract is 34.30%. Table 1 shows the contents of polyphenols, flavonoids, and tannins in the methanolic extract of *Myrtus communis* L. The data clearly show considerable amounts of all three.

### 2.2. GC-MS Analysis

GC-MS is a key technological platform that is well established for providing a somewhat clearer picture of pharmaceutical ingredients [39]. The chemical profile of the investigated plant extract, which is referred to as the “fingerprint” of traditional Algerian medicine, can be clearly reflected by a chromatogram.

**Figure 1:**
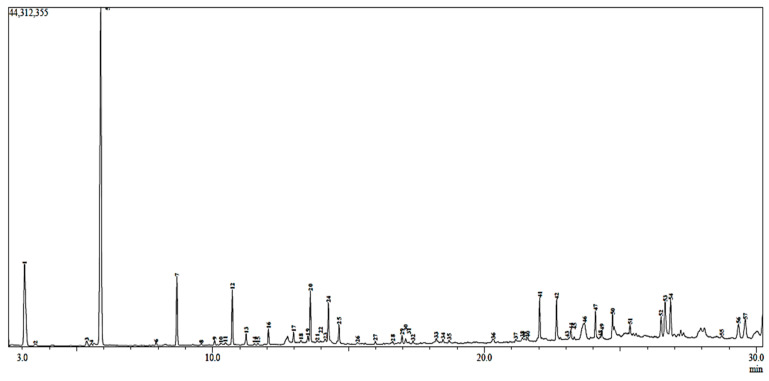
Representative GC-MS chromatogram of *Myrtus communis* L. methanolic extract. Peak numbers correspond to compound numbers in Table 2.

The results of the GC-MS analysis of MMEx are presented as a chromatogram in Figure 1. The compounds are listed according to their mass/charge ratio (*m*/*z*) in Table 2. A total of 57 bioactive compounds were putatively identified. The predominant compound in myrtle extract was 1,8 cineol (33.80%). Alpha-pinene constituted 10.06%, followed by linalool (4.83%) and elaidic acid, and isopropyl ester (4.21%). The extract of myrtle contains flavonoids, including pinostrobin chalcone, and phenolic acid derivatives, including cinnamyl cinnamate.

### 2.3. Antioxidant Activity

The ability to remove free radicals by MMEx is dependent on the polyphenolic content. It was clearly observed that the presence of a high phenolic and flavonoid content was in harmony with the antioxidant activity exerted by MMEx in all antioxidant tests. However, in high concentrations (100–200 µg.mL^−1^), there was no significant change in inhibition, probably because the critical concentration of phenols is sufficient to achieve the desired quenching power, and the over-saturation concentration has no effect on the antioxidant level.

**Figure 2:**
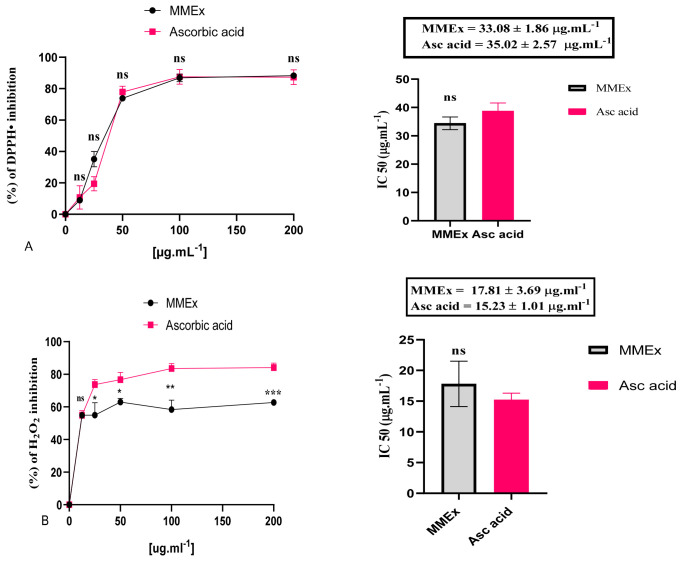
Antioxidant properties of MMEx and ascorbic acid (reference) by (**A**) DPPH and (**B**) H_2_O_2_, and their IC_50_ values (b, d, f, h, and j, respectively). Data are expressed as mean ± SEM (n = 3). Ns, no significant difference. * *p* < 0.05, ** *p* < 0.01, *** *p* < 0.001. (*t* test) using GraphPad Prism 8 for Microsoft Office 10.

During the experiment, a change in DPPH intensity was visually apparent, as the color changed from deep violet to yellow. Inhibition increased from 8.97 ± 1.63% at 12.5 µg.mL^−1^ to 88.25 ± 0.40% at 200 µg.mL^−1^ (Figure 2A), with an IC_50_ of 33.08 ± 1.86 µg.mL^−1^, which was comparable to ascorbic acid (IC_50_ of 35.02 ± 2.57 µg.mL^−1^) with no significant difference.

Furthermore, MMEx exhibited a strong H_2_O_2_ radical scavenging ability compared to the reference molecule, with inhibition increasing from 54.89 ± 1.28% to 62.70 ± 1.22% and from 54.90 ± 2.79% to 84.16 ± 2.73% for MMEX and ascorbic acid, respectively, at 12.5 to 200 (µg.mL^−1^) concentration (Figure 2B). The IC_50_ values of MMEx and ascorbic acid were 17.81 ± 3.69 and 15.23 ± 1.01 µg.mL^−1^, respectively.

**Figure 3:**
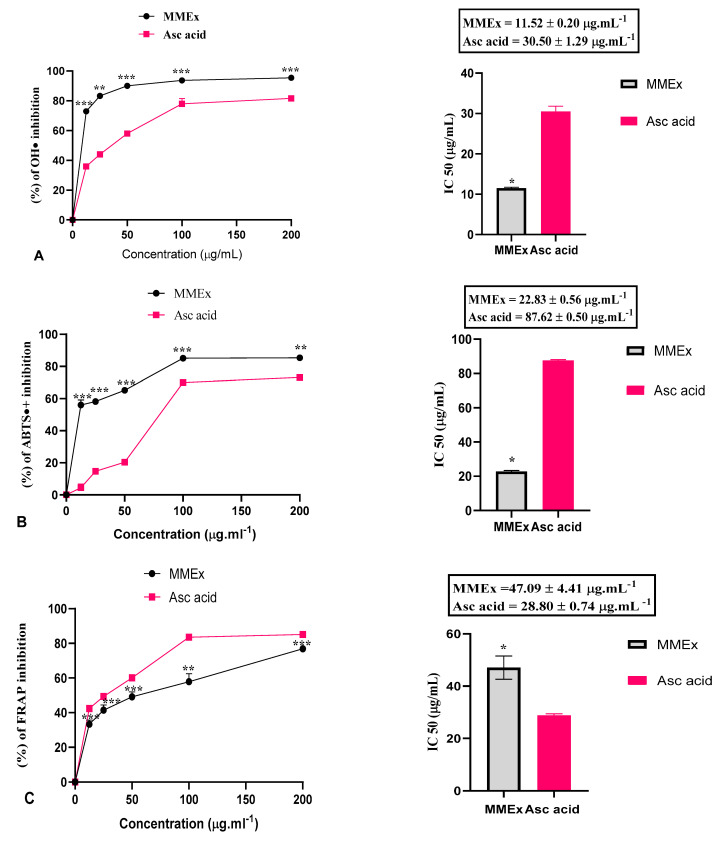
Antioxidant properties of MMEx and ascorbic acid (reference) by (**A**) hydroxyl radical, (**B**) ABTS, and (**C**) FRAP and their IC_50_ values. Data are expressed as mean ± SEM (n = 3). ns, no significant difference. * *p* < 0.05, ** *p* < 0.01, *** *p* < 0.001 (*t*-test) using GraphPad Prism 8 for Microsoft Office.

As shown in Figure 3A, the hydroxyl radical OH-neutralizing test showed that MMEx had significantly higher (*p* < 0.05) antioxidant activity, with an IC_50_ value of 11.52 ± 0.20 µg.mL^−1^, which is 2.65 times higher compared to the reference molecule (30.50 ± 1.29 µg mL^−1^).

The blue-green ABTS•+ solution turned pale yellow and then colorless. MMEx reduced the ABTS•+ species in a dose-dependent manner, with inhibition increasing from 56 ± 3.18% at 12.5 µg.mL^−1^ to 85.27 ± 0.87 at 200 µg.mL^−1^ (Figure 3B). This excellent power in capturing ABTS•+ species was reflected by an IC_50_ of 22.83 ± 0.56 µg.mL^−1^, which is statistically significant (*p* < 0.05) and 3.9 times higher compared to the reference standard ascorbic acid (87.62 ± 0.50 µg mL^−1^).

Figure 3C illustrates the capacity to transform a ferric ion (Fe^3+^) into a ferrous ion (Fe^2+^), creating a chromogenic complex. The samples underwent a change in color from yellow to pale/light green or Prussian blue in the solution, and a high level of FRAP-capturing activity by MMEx was noted, as expected. MMEx displayed 77.91 ± 0.54% inhibition against ascorbic acid and 85.09 ± 0.34% at the highest tested concentration (200 µg.mL^−1^). The IC_50_ value of MMEx and the standard was 47.09 ± 4.41 µg and 28.80 ± 0.74 µg.mL^−1^, respectively.

### 2.4. Anti-Inflammatory Activity

#### 2.4.1. In Vitro: Inhibition of BSA Denaturation

**Figure 4:**
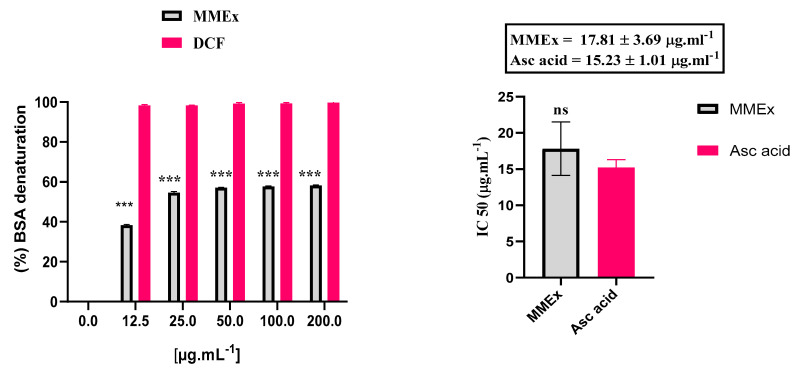
Inhibition of heat-induced BSA denaturation. Data are expressed as mean ± SEM (n = 3). DCF was used as positive control. Mean values of samples showing significant differences compared to control (untreated 5% BSA water solution). *** *p* < 0.001 (*t*-test) using GraphPad Prism 8 for Microsoft Office. IC_50_ analysis performed using GraphPad Prism 8.

As a part of the inquiry into the mechanism of anti-inflammatory action, the potential of MMEx to suppress the denaturation of proteins was explored. The inhibitory effects of various concentrations of MMEx with the highest polyphenol content on protein denaturation are depicted in Figure 4. A concentration-dependent inhibition of BSA denaturation via MMEx in the range of 12.5–200 μg. mL^−1^ was noted. MMEx at 200 mg.kg^−1^ achieved a highly significant (*p* < 0.001) inhibition of BSA denaturation of (58.27 ± 0.19%) in a dose-dependent manner. The anti-inflammatory activity of MMEx in vitro was comparable to that of the well-known nonsteroidal anti-inflammatory drug diclofenac (DCF). A significant difference in the inhibition of thermally induced protein denaturation was observed with MMEx, reflected by an IC_50_ value of 23.86 ± 0.31 µg.mL^−1^, which is 2.43 times lower than the IC_50_ of DCF (9.82 ± 3.14 µg.mL^−1^).

#### 2.4.2. In Vivo: Carrageenan-Induced Paw Edema in Rats

**Figure 5:**
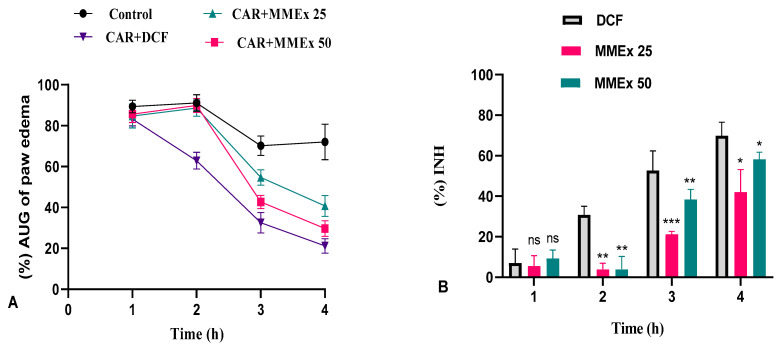
(**A**) Influence of MMEx (25 and 50 mg/kg) on Car-induced paw edema. Data represent percentage of increased paw edema (mean ± SEM) in different groups. (**B**) Inhibition of paw edema in rats treated with myrtle methanolic extract. Ns, no significant difference. * *p* < 0.05, ** *p* < 0.01, *** *p* < 0.001 (*t* test) using GraphPad Prism 8 for Microsoft Office. IC_50_ analysis performed using GraphPad Prism 8.

Paw edema in the experimental groups is shown in Figure 5. A significant increase in paw volume was observed in all groups that received sub-plantar injections of 1% Car solution compared to the control group 2 h after injection, and the increase was sustained throughout the experiment. The increase in paw volume was lower in rats that received DCF and the investigational polyphenolic fraction of MMEx at both doses (25 and 50 mg.kg^−1^) compared to the untreated positive control (Car). The inhibitory effect of the lower dose (25 mg/kg) of MMEx lasted for a shorter time than the effect of the higher dose (50 mg/kg). At the end of the experiment, the inhibitory effect of the highest dose of MMEx (50 mg.kg^−1^) did not differ significantly from the effect of DCF. The lower dose of MMEx (25 mg/kg) inhibited paw edema to a lesser extent than DCF and the higher dose of MMEx. The reference drug, DCF, showed a biphasic pattern and its highest inhibition (69.83 ± 6.74%; mean ± SEM) occurred at 4 h (*p* < 0.01). MMEx showed a similar inhibition of paw edema, with the highest inhibition recorded as 58.26 ± 3.41% at the forth h (*p* < 0.01).

#### 2.4.3. In Silico Study

The binding energy between molecules determines the effect of molecular docking. Lower molecular docking-binding energy represents higher binding affinity. When the binding energy is <5 kcal/mol, the receptor and ligand have relatively good binding properties.

The anti-inflammatory mechanism of the extract as determined by in vivo and in vitro experiments was demonstrated by the in silico analysis.

- Drug-likeness of Compounds

As shown in Supplementary Table S1, the compounds of the extract were evaluated for their ability to serve as active oral drugs. Out of the 57 compounds from MMEx, 27 were considered to be drug-like compounds while the remaining 30 were not included in further analyses, since they failed the drug-likeness test.

- Effect on pro-inflammatory targets: Cox-2, IL-1β, NF-κB, PLA2, and TNF-α

**Figure 6:**
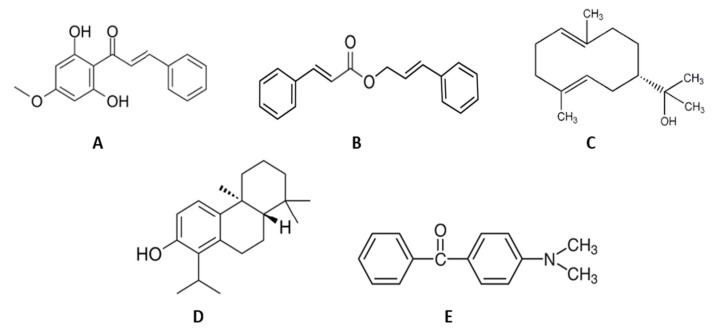
Chemical structure of five compounds from myrtle methanolic extract that presented good binding affinity against key anti-inflammatory proteins (Cox-2, IL-1β, NF-κB, PLA2, and TNF-α). (**A**) pinostrobin chalcone; (**B**) cinnamyl cinnamate; (**C**) hedycaryol; (**D**) totarol; (**E**) p-dimethylaminobenzophenone.

The 27 compounds were virtually screened against five key anti-inflammatory proteins: Cox-2, IL-1β, NF-κB, PLA2, and TNF-α. Among the 27 compounds, 5 were observed to have good binding affinity based on the docking scores: pinostrobin chalcone, cinnamyl cinnamate, hedycaryol, totarol, and p-dimethylaminobenzophenone-. The outcome is summarized in Table 3. The chemical structures of the five compounds are presented in Figure 6.

Pinostrobin chalcone (−8.8 kcal/mol) and cinnamyl chalcone (−9.5 kcal/mol) were found to have higher binding scores than the other compounds and diclofenac when docked against the active site of Cox-2. For IL-1β, hedycaryol (−6.2 kcal/mol) and totarol (−6.2 kcal/mol) stood out from the other compounds after docking. Cinnamyl cinnamate (−6.5 kcal/mol) and totarol (−7.1 kcal/mol) were found to have good binding scores with NF-κB. However, totarol (−8.1 and −7.0 kcal/mol) and p-dimethylaminobenzophenone (−8.5 and −6.5 kcal/mol) were found to be good binders of PLA2 and TNF-α, respectively.

**Figure 7:**
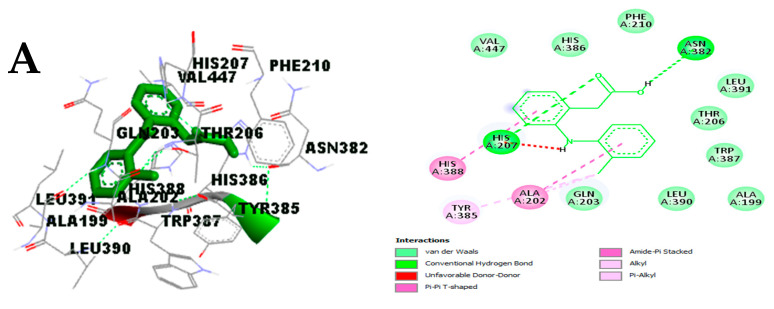

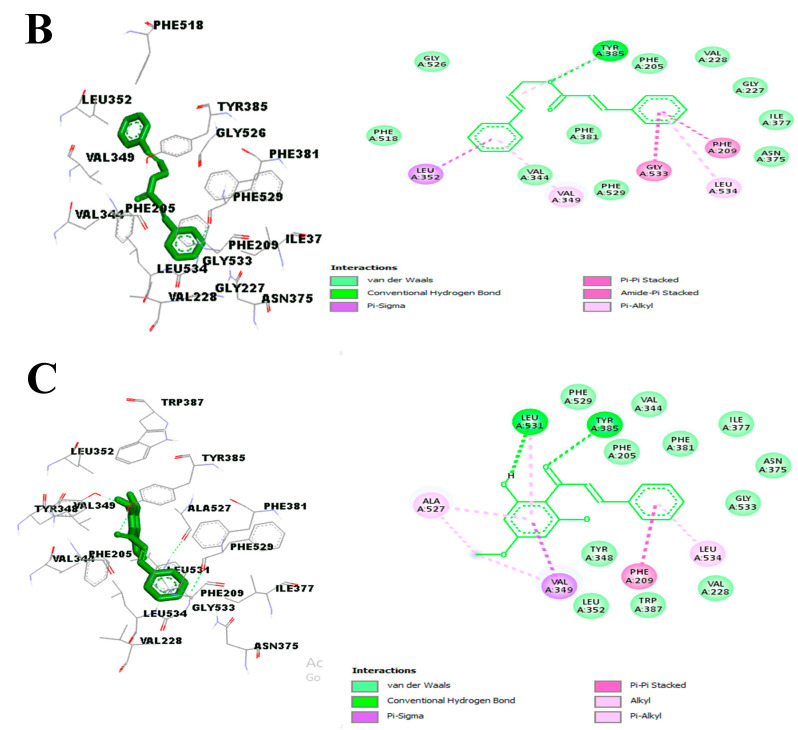
Binding interaction of COX-2 with (**A**) diclofenac, (**B**) cinnamyl cinnamate, and (**C**) pinostrobin chalcone. Purple indicates pi-sigma, violet indicates pi-pi stacked, and light pink indicates alkyl/pi-alkyl.

**Figure 8:**
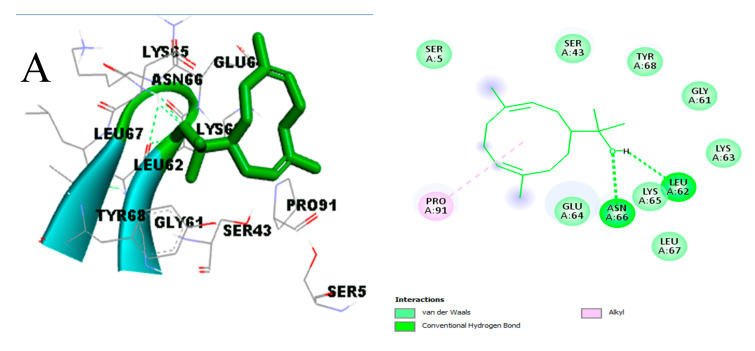

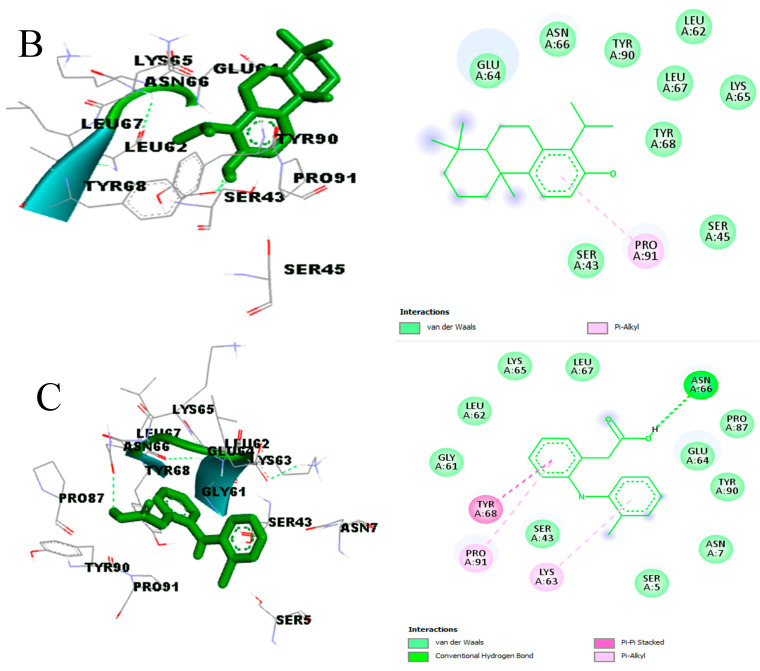
Binding interaction of IL-1β with (**A**) diclofenac, (**B**) hedycaryol, and (**C**) totarol.

**Figure 9:**
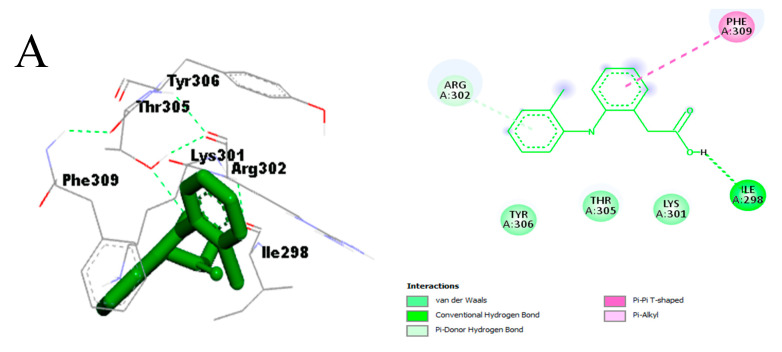

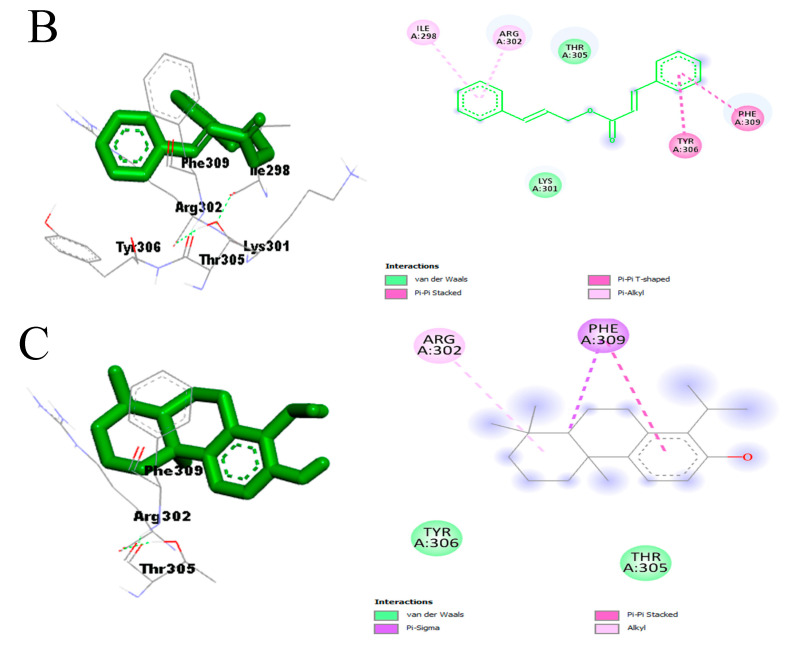
Binding interaction of NF-κB with (**A**) diclofenac, (**B**) cinnamyl cinnamate, and (**C**) totarol.

**Figure 10:**
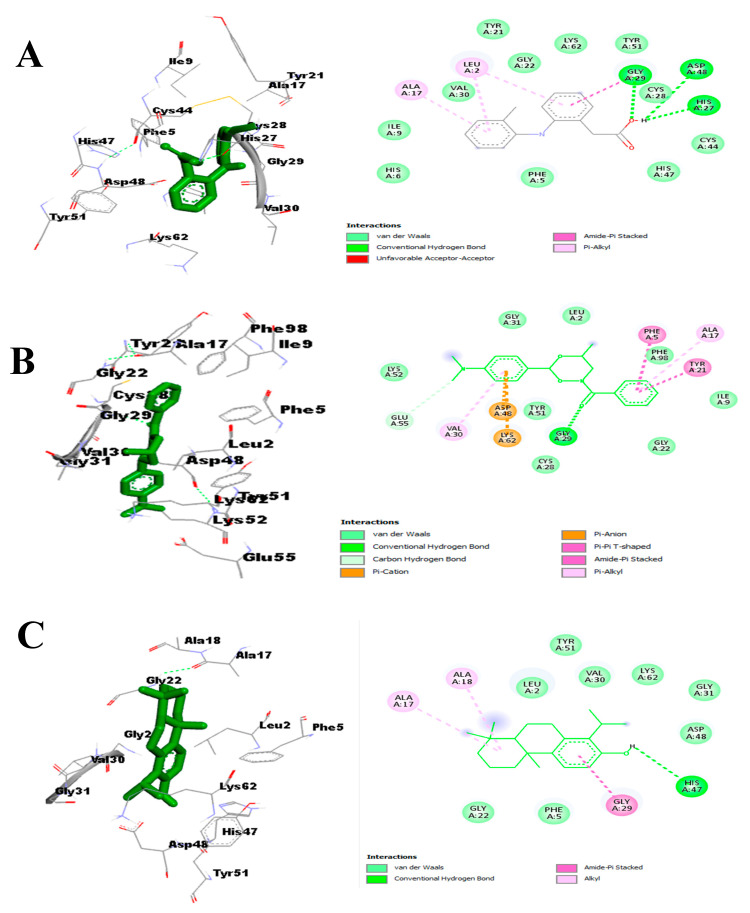
Binding interaction of phospholipase A2 with (**A**) diclofenac, (**B**) p-dimethylaminobenzophenone, and (**C**) totarol.

**Figure 11:**
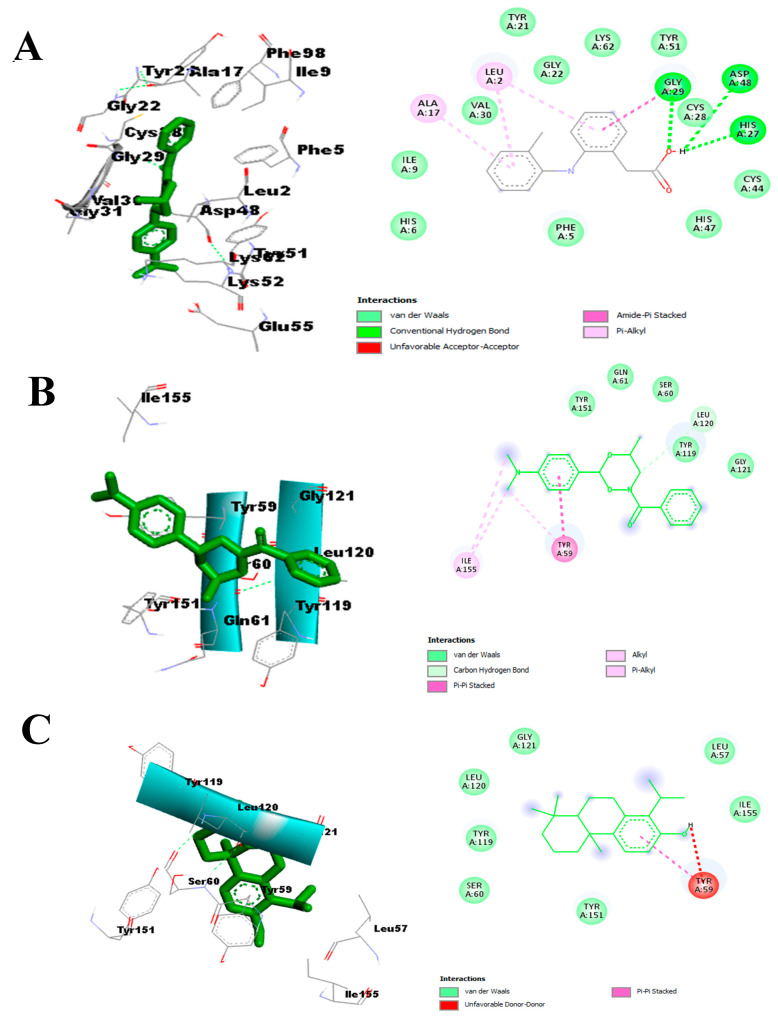
Binding interaction of TNF-α with (**A**) diclofenac, (**B**) p-dimethylaminobenzophenone, and (**C**) totarol.

The molecular interaction analysis of compounds with diclofenac that were found to have good binding with the five targets is presented in Figure 7, Figure 8, Figure 9, Figure 10 and Figure 11. The analysis shows how the functional groups of the compounds interact. These interactions are critical to the stability and binding of a given compound in the binding pocket of a given protein. 

The type of interaction observed with diclofenac, cinnamyl cinnamate, and pinostrobin chalcone after visualization were mainly hydrogen bonds and hydrophobic and pi-interactions with the binding site of Cox-2 (Figure 7A–C). Hydrogen bonds were established between the atoms of cinnamyl cinnamate and pinostrobin chalcone and side chains of Tyr385 and Leu531 while those of diclofenac were with His207 and Asn382 (Figure 7).

The predominant amino acids interacting with the atoms of the two hit compounds and diclofenac via hydrophobic and pi-interactions are shown in light green (van der Waals). Following visualization, hydrogen bonds, hydrophobic interactions, and pi-interactions with the IL-1β active site were the main types of molecular interaction seen with diclofenac, hedycaryol, and totarol (Figure 8A–C). No hydrogen bonds formed between the atoms of totarol and the amino acid side chains in the active site of IL-1β. Hydrogen bonds were observed between the atoms of diclofenac, hedycaryol, totarol, and side chains of Asn66 and Leu62. The predominant amino acids interacting with the atoms of the two hit compounds and diclofenac via hydrophobic and pi-interactions are shown in light green (van der Waals), purple (pi-sigma), and violet (pi-pi stacked) and light pink (alkyl/pi-alkyl) (Figure 8).

The molecular interactions observed with diclofenac, cinnamyl cinnamate, and totarol after visualization were mainly hydrogen bonds (diclofenac, Ile298) and hydrophobic and pi-interactions with the active site of NF-κB (Figure 9A–C). No hydrogen bonds formed between the atoms of cinnamyl cinnamate, totarol, and the amino acid side chains in the active site of NF-κB. The prominent amino acids interacting with the atoms of the two hit compounds and diclofenac via hydrophobic and pi-interactions are shown in light green (van der Waals), purple (pi-sigma), violet (pi-pi stacked), and light pink (alkyl/pi-alkyl) (Figure 9).

The chemical interactions with diclofenac, p-cimethylamino benzophenone, and totarol were primarily hydrogen bonds and hydrophobic and pi-interactions with the phospholipase A2 active site (Figure 10A–C). Hydrogen bonds formed between the atoms of p-dimethylaminobenzophenone, totarol, and the amino acid side chains of Gly29, Asp48, His27, and Glu55 in the active site of phospholipase A2. The predominant amino acids interacting with the atoms of the two hit compounds and diclofenac via hydrophobic and pi-interactions are shown in light green (van der Waals), purple (pi-sigma), violet (pi-pi stacked), orange (pi-charges), and light pink (alkyl/pi-alkyl) (Figure 10).

The molecular interaction fingerprints observed with diclofenac, p-dimethylaminobenzophenone, and totarol after visualization were majorly hydrogen bonds, hydrophobic and pi-interactions with the active site of TNF-α (Figure 11A–C). The hydrogen bonds formed between the atoms of p-dimethylaminobenzophenone, totarol, and the amino acid side chains of Gly122, Gly121, Leu120, and Tyr59 in the active site of TNF-α. The predominant amino acids interacting with the atoms of the two hit compounds and diclofenac via hydrophobic and pi-interactions are shown in light green (van der Waals), purple (pi-sigma), violet (pi-pi stacked), and light pink (alkyl/pi-alkyl) (Figure 11).

### 2.5. ADMET Results

The admetSAR web platform (http://lmmd.ecust.edu.cn/admetsar2; accessed on 25 June 2023) was used to predict the absorption, distribution, metabolism, and toxicity (ADMET) of the reference drug (DCF) and the hit compounds (cinnamyl cinnamate, pinostrobin chalcone, hedycaryol, totarol, and methanone [4-methyl-6-(4-dimethylamino)-1,5,2-dioxazinan-3-yl] (phenyl)-). In the absorption class, caco-2 permeability, human intestinal absorption, human oral availability, p-glycoprotein inhibition, and substrates were predicted. All were predicted to permeate caco-2, which is absorbed by the human intestine. Only DCF and hedycaryol are orally bioavailable. Methanone [4-methyl-6-(4-dimethylamino)-1,5,2-dioxazinan-3-yl] (phenyl)-) was predicted to inhibit p-glycoprotein, and none of the compounds can serve as a substrate for p-glycoprotein.

The distribution of these molecules was predicted via two properties, PPB and BBB. DCF, pinostrobin chalcone, and totarol might be able to reach target sites in high to moderate doses, as their ability to bind to plasma proteins was predicted to be high. Only pinostrobin chalcone cannot permeate the blood–brain barrier (BBB). All compounds were predicted to be localized in the mitochondria except hedycaryol, which is localized in the lysosome.

The metabolism of these molecules via the human liver was predicted in order to ascertain whether they can cause harm or not. Diclofenac, cinnamyl cinnamate, pinostrobin chalcone, totarol, and methanone [4-methyl-6-(4-dimethylamino)-1,5,2-dioxazinan-3-yl] (phenyl)-) were predicted to inhibit cytochrome P450 1A2. Also, cinnamyl cinnamate and pinostrobin chalcone were predicted to inhibit cytochrome P450 2C19. The inhibitors of cytochrome P450 2C9 were predicted to be diclofenac and pinostrobin chalcone. Only pinostrobin chalcone was predicted to inhibit cytochrome P450 3A4.

DCF and totarol were predicted to be substrates for cytochrome P450 2C9. Totarol was also predicted to be a substrate for cytochrome P450 2D6 and 3A4. Methanone [4-methyl-6-(4-dimethylamino)-1,5,2-dioxazinan-3-yl] (phenyl)-) was likewise predicted to be a substrate for cytochrome P450 3A4.

All compounds were catalyzed by UGT except pinostrobin chalcone, as predicted by admetSAR. Toxicity was evaluated based on acute oral toxicity, Ames mutagenesis, carcinogenicity, and human hepatotoxicity. None of the compounds were predicted to be carcinogenic, although diclofenac, pinostrobin chalcone, hedycaryol, and methanone [4-methyl-6-(4-dimethylamino)-1,5,2-dioxazinan-3-yl] (phenyl)-) were predicted to be hepatotoxic. Cinnamyl cinnamate and hedycaryol were predicted to have Ames mutagenicity. The acute oral toxicity (kg/mol) was also predicted (Table 4).

## 3. Discussion

### 3.1. Phytochemical Analysis

The good ethnopharmacological reputation of *Myrtus communis* L. is partly responsible for the choice of this plant for investigation. The presence of various bioactive compounds has justified its use from ancient times for the treatment and management of inflammation and accompanying symptoms. Researchers have confirmed that leaf extracts have a better antioxidant effect compared to myrtle berry extracts due to the galloyl derivatives, flavonols, and flavonol derivatives [30], which informed the use of leaves in our work.

The resulting viscous mass of MMEx had a dark green color and a fruity, herbaceous aroma. The extraction yield was 34.30% (*wt*/*wt*) of dry material on average, which was similar to the 35.56% reported in extract from Zaccar, Ain Defla, Algeria [40]. Our results partially agree with those of other reports, in which the yield was 30.6% [41]. In contrast, our yield was lower than what was previously obtained from Zakynthos, a Greek island, with percentages ranging between 43.4 and 59.5% [42], while it was noted to be higher than the 28.66% reported in a study by Hayder et al. for leaves harvested from northeast Tunisia [43]. A lower yield of 10.50% was reported in another study [44].

Our findings indicate that the phenolic content (415.85 ± 15.52 mg GAE/g DW) was higher than the flavonoid content (285.8 ± 1.64 mg QE/g DW). This is in agreement with the results reported in Giza, Egypt, where the TPC of myrtle alcoholic extract was 472.47 ± 3.73 mg GAE/g and the TFC was 281.15 ± 21.88 mg QE/g, which is within the range obtained in our study. Gardeli and co-workers reported a high content of phenolics from the methanolic extract of myrtle leaves ranging between 307 ± 7.4 and 373 ± 0.5 mg GAE/g [42]. Bouaziz et al. [45] reported a TPC of 260.44 ± 2.52 mg GAE/g and a TFC of 26.77 ± 0.46 mg QE/g, which are lower than our results [45]. A low TPC of 33.67 mg GAE/g was reported in a study by Aidi Wannes and his research team in Tunisia [46]. Our content was higher when compared to that obtained with MMEx from Turkey, which had TPC and TFC values of 190.85 ± 0.73 mg GAE/g and 43.97 ± 2.07 mg QE/g, respectively [44]. Tannins are secondary antioxidants that have the capacity to bind metal ions like Fe^2+^, interfere with one of the stages in the Fenton reaction, and slow down the oxidation process in addition to their anti-inflammatory, antitumor, and antimicrobial properties [47].

The total phenolic and flavonoid contents do not give a complete picture of the quality and quantity of the phenolic and flavonoid constituents, and GC-MS analysis can provide the most helpful information on individual phenolic compounds [48]. To our knowledge, MMEx has not been analyzed by GC-MS in previous studies. For this reason, comparisons were made with other species of the Myrtaceae family. Herein, 57 compounds identified in MMEx, constituting 71% of the whole extract, are listed in the order of their column elution time in Table 3, and they are already known for several biological activities. The predominant compound, 1.8-cineole (33.8%), which possesses important anti-inflammatory activity, is widely used in the cosmetic and pharmaceutical industries [49]. The second main substance, α-pinene (10.06%), was noted to have antioxidant, anticancer, and anti-inflammatory activities. Linalool (4.83%) has also been reported to have antioxidant, antimicrobial, anti-inflammatory, antitumor, and insecticidal properties [25,28].

It is well known that several factors affect the yield of extraction, the kinetics of phenolic compounds and flavonoids released from the solid matrix, and the composition quality of the extract, such as the part of the plant used and its geographical origin, the period of harvest, and the extraction procedure [50]. In general, the yield of the extraction process is directly influenced by the polarity indices, types, and concentrations of organic solvents with the extraction time [51,52].

Phytochemical analysis vividly proved that our extract is a rich source of phenolic compounds; their presence in high concentrations explains the higher level of antioxidant and anti-inflammatory activities. In this scope, we decided to unravel the biological properties of MMEx using in vitro, in vivo, and in silico approaches.

### 3.2. Antioxidant Activity

The scientific community has shown heightened interest in exploring medicinal plants, and this spike in interest can be attributed to the increasing number of publications focused on the topic, reflecting a growing recognition of their biological value, which represents a new wave of secure therapies for the prevention, management, and treatment of diseases involving oxidative stress and inflammation [53]. It is believed that a single antioxidant property model cannot precisely mirror the mechanism of action of an investigated plant [54,55]; thus, modeling is frequently performed using combinations of parameters, with a focus on the kinetics of reactions [56].

In the DPPH radical scavenging test, MMEx presented a radical quenching percentage greater than 80% at the highest tested concentration (200 µg.mL^−1^), with an IC_50_ of 33.08 ± 1.86 µg.mL^−1^, indicating statistically non-significant activity when compared to ascorbic acid (IC_50_ of 35.02 ± 2.57 µg.mL^−1^). This finding agrees with another study in which the methanolic extract of Italian myrtle was found to have an inhibition percentage greater than 90% at a concentration of 50 µg.mL^−1^ by DPPH testing [57]. Our results are also in accordance with those reported in a study by Gardeli et al., in which MMEx effectively removed DPPH with an IC_50_ ranging between 9.54 ± 0.93 and 17.1 ± 0.78 µg.mL^−1^ [42]. Excellent scavenging activity of myrtle extract, with an IC_50_ value of 6 µg.mL^−1^, was also reported [45].

H_2_O_2_ is the primordial source of OH^•^. In the presence of transition elements in reduced form (e.g., Fe^2+^), the Fenton and Haber–Weiss reactions lead to the formation of the most harmful radical OH^•^ with a limited lifespan. Its half-life in the biological system is about 1 ns, and it reacts quickly with organic molecules with rate constants of 109–1010 M^−1^ s^−1^. OH^•^ is involved in various deadly diseases such as cancer by its ability to activate oncogenes, especially C-Raf-1 and K-ras [57,58]. Hydroxyl radicals are especially harmful because of their ability to reduce disulfide bonds in proteins, leading to unfolding and unnatural refolding into abnormal configurations [59]. In these circumstances, removing hydrogen peroxide is very important, since this radical is very reactive with cell machinery [60]. In our study, MMEx displayed strong H_2_O_2_ and hydroxyl radical scavenging activity in a content-dependent manner, reflected by IC_50_ values of 17.81 ± 3.67 and 11.52 ± 0.20 µg/mL, respectively. The extract of *M. communis* considerably decreased the degradation of Fe^2+^ via H_2_O_2_ compared to vitamin C, with an IC_50_ value of 275 µg.mL^−1^ [45]. Our result is more important than the one in a study by Benchikh et al. [61], where MMEx reduced hydroxyl radicals with an IC_50_ of 140 μg/mL.

The ABTS and FRAP scavenging activity of MMEx was reflected by IC_50_ values of 22.83 ± 0.56 and 47.09 ± 4.41 µg/mL, respectively. A similar trend was observed for the ABTS^•+^ removal capacity of MMEx, where the IC_50_ was 13.6 μg.mLl^−1^ [62]. In another study, the authors reported that myrtle extract effectively scavenged ABTS^•+^, with an IC_50_ of 4.8 µg.mL^−1^ [45].

The antioxidant properties of plants are frequently associated with the polyphenolic content of aromatic amines, phenolic acids, essential oils, flavonoids, proanthocyanins, and bioactive compounds [63,64], which can act as electron donors and react with free radicals to transform them into more stable products and prevent radical chain reactions [65]. The higher antioxidant power of MMEx may support the substantial amount of cell-reinforcing chemicals present in our extract [66]. According to the literature, a phenolic content higher than 20 mg GAE/g can be considered very high and sufficient to ensure biological prevention against oxidants [67]. When ROS combine with the extract, a chain of events is initiated and reactive phenoxy radicals are formed when phenol groups accept electrons, which again combine with ROS and act as reducing agents, singlet oxygen quenchers, and hydrogen-donators, interrupting the oxidation action chain [68]. The ability of polyphenols to rescue the redox balance, their structural motifs, such as the carboxylic acid group, and the presence of hydroxy and methoxy groups with lower bond dissociation energy are responsible for their extensive biological activities [52,69,70].

### 3.3. Anti-Inflammatory Activity

#### 3.3.1. In Vitro: Inhibition of BSA Denaturation

Given the plant’s strong antioxidant effect under examination and the close correlation between inflammation and oxidative stress, anti-inflammatory activity was also investigated in the present study using an in vitro model of protein denaturation, which, given its features of being simple, quick, and inexpensive, represents the gold standard for the evaluation of this parameter [71].

Inflammation has been known to be linked with protein denaturation, membrane alteration, increased vascular permeability, and pain. Chronic inflammation has been closely associated with tumor progression [71]. Denaturation causes changes in the physiochemical properties of protein, which can be caused by inflammatory agents [72]. Some external factors, including stress and chemicals, can induce denaturation, leading to the loss of the highly ordered quaternary, tertiary, and secondary structures and the breakage of many weak bonds, including hydrogen bonds responsible for the native structure of protein; this is one of the significant characteristics of inflammation. It was suggested that bioactive substances that are capable of inhibiting the production of heat-induced protein denaturation can be used as therapeutic anti-inflammatory drugs [55]. The search for highly biocompatible drugs from plants is an ongoing research effort. Therefore, the anti-inflammatory activity of MMEx was investigated by determining its potential to protect against protein denaturation. MMEx was found to be effective against heat-induced BSA denaturation. DCF displayed a maximum inhibition of 99.73 ± 0.27% at 200 µg/mL (Figure 3A) [73]. The data suggest that MMEx is protective against heat-induced albumin denaturation. Further, it was found that an increased concentration of MMEx increased the inhibition of denaturation. Extracts at a concentration of 200 µg/mL showed good anti-inflammatory potential by inhibiting heat-induced albumin denaturation by 58.27%.

#### 3.3.2. In Vivo: Car-Induced Paw Edema

Investigations into the anti-inflammatory potency of *Myrtus communis* are limited. Only a few research reports suggest that MMEx can reduce Car-induced paw edema in animals, although the precise mechanism is not entirely addressed. Edema is one of the obvious indicators of inflammation and a key factor to take into account when assessing the anti-edematogenic and anti-inflammatory properties of a substance [74]. Carrageenan is an acknowledged pro-inflammatory agent that has been used to initiate acute inflammatory episodes upon injection into the footpad of rodents, and it well recapitulates the pathogenesis characteristics [75,76]. Carrageenan has been found to resist biodegradation due to its persistence within Kupffer cells for several months [9]. It is characterized by the excessive generation of free radicals and inflammatory cytokines, which results in the elevated inflammation and sensitivity of axonal terminals to pain [76]. Carrageenan causes acute inflammation via a biphasic pattern: (1) the early phase, lasting from 1 to 2.5 h, with the release of mediators including histamine, serotonin, 5-hydroxytryptamine, kinin, and bradykinin; and (2) the late phase, lasting from 2.5 to 6 h, with an elevation in PGs and COX-2, neutrophil infiltration, and ROS production [77].

In our experiment, significant paw edema in the Car-injected animals was observed in all groups. Pretreatment with MMEx inhibited the second phase of Car-induced edema in a dose-dependent manner relative to the positive control group. Two hours after Car administration, there was no significant difference in the inhibition of paw edema between animals pretreated with DCF and the two doses of MMEx. DCF inhibits PLA2, a key mediator of several processes in the inflammatory cascade, interrupting this cycle [78]. It was reported that NSAIDs inhibit only the late phase when PGs and Cox-2 enzymes are detectable [79]. The same result was reported in a study by Amira et al. [80], in which myrtle extract had the highest inhibitory activity against paw edema induced by Car (60%) after 3 h [81]. These data are consistent with the findings of other studies, in which alcoholic extract from myrtle leaves reduced edema by 56.6% compared to indomethacin (64.3%) [81]. The anti-inflammatory effect of *Myrtus communis* was investigated in several studies, and oligomeric nonprenylated acylphloroglucinols isolated from myrtle were determined to be potent inhibitors of eicosanoid biosynthesis [10]. Oral administration of hydroalcoholic extract from *Lampaya medicinalis Phil.* in rats resulted in reduced paw edema, which was lower compared to our result [82]. A similar trend was found in a xylene-induced ear edema model; Hosseinzadeh et al. [28] found that ethanolic extracts of the aerial parts of *M. communis* L. (50 mg.kg^−1^) effectively and significantly reduced inflammation through the proliferative phase with an inhibition of 59.8% [28]. The ethanolic extract of myrtle at a dose of 44.4 mg/kg was reported to inhibit edema by 34.7% [83].

Anti-inflammatory activity was evaluated by measuring 6-keto-prostaglandin F1 and [3H]-arachidonic acid metabolite production in keratinocytes stimulated for inflammation, and the results showed that the extract significantly decreased all metabolite production from the pathway [25]. When administered intraperitoneally at a dose of mg/kg, *M. communis* reduced the development of Car-induced mouse paw edema in a dose-dependent manner [84]. Myrtucommulone (MC), semimyrtucommulone (S-MC), and nonprenylated acylphloroglucinols in the leaves of *M. communis* were found to potently suppress eicosanoid biosynthesis by directly inhibiting cyclooxygenase-1 and 5-lipoxygenase in vitro and in vivo [85]. However, in another report, myrtle was believed to inhibit the cell-free mPGES-1-mediated conversion of prostaglandin H2 (PGH2) to PGE2 [86]. Seed extract (50 mg.kg^−1^ for 2 months) decreased the plasma levels of inflammatory cytokines TNF-α, IL-8, IL-6, and IL-1β as well as erythrocyte concentration, ROS level, and lipid peroxidation, and fortified the activity of the main antioxidant enzymes [87]. It was reported that phenolic compounds are similar to NSAIDs in their ability to decrease the chemical mediators of inflammation [75]. We hypothesize that the increased and more varied content of bioactive substances in MMEx explains the higher anti-inflammatory activity by diminishing TNF-α, IL-1β, and COX targets or by blocking the NF-κB pathway and its messengers. Flavonoids are also known to inhibit NF-κB-dependent signaling, which is required for angiogenesis, survival, and the proliferation of cancer cells [70]. There is still a debate regarding the biological activities of many natural extracts and how to use them in the right way [88].

Obviously, we should take into account the short duration of the experiment and the single exposure of the tissue to the inflammatory factor. The mechanism of action of plant extracts cannot be quickly judged; it depends on their chemical composition, and it is notoriously difficult to attribute biological activity to a single mechanism rather than a cascade of reactions. MMEx can cure inflammation through a synergistic effect with multicomponent, multi-target, and multi-pathway approaches, and to verify the above hypothesis, we used in silico tools. Comprehending the deep molecular mechanisms could enable the design of precise roadmaps that can lead to more successful therapeutic solutions [89].

#### 3.3.3. In Silico Study

Prior to the molecular docking run, 57 compounds from the plant extract with anti-inflammatory properties were subjected to drug-likeness analysis. This analysis ensured the removal of phytocompounds with poor pharmacokinetic parameters from the drug pipeline, thus saving time and cost. Using the SwissADME web tool, we found that 27 of the 57 screened compounds could potentially be used as drugs based on multiple scoring schemes.

The binding affinity between the docked compounds (ligands) and target proteins in this study were influenced by non-covalent interactions such as hydrogen bonds, hydrophobic bonds, and pie-stack interactions (Figure 6, Figure 7, Figure 8, Figure 9 and Figure 10). Many researchers have confirmed that binding affinity will be higher (binding energy will be lower) if ligands are able to form hydrophobic interactions with appreciable numbers of hydrophobic amino acid residues in the binding site of a target protein [90,91]. Stojanovi and Zari [92] reported the significance of hydrophobic interactions in many systems with strong intermolecular forces [92]. This may be responsible for the appreciable binding affinity of the active compounds docked against the selected proteins in this study. Notably, the impact of hydrogen bonds on the stabilization of molecular interactions between ligands and proteins should not be downplayed due their critical role in enzyme catalysis and protein–substrate and protein–inhibitor complexes, including the structural stability of many biological molecules [89,92]. To this end, the analysis of molecular interactions exhibited by the active compounds assessed in this study as judged by their binding energy, hydrogen bonding, and hydrophobic interactions with the surrounding amino acids in the selected protein targets revealed the positive impact of their ligand–protein complex status, which may enhance the mechanism of action of the essential oil in ameliorating chronic inflammation. The logic behind performing the absorption, distribution, metabolism, excretion, and toxicity (ADMET) screening of any chemical compound within the human body is that it can determine the pharmacological and pharmacodynamic properties of candidate drug compounds within the biological system.

Adsorption, distribution, metabolism, excretion, and toxicity (ADME/Tox) screening is an increasingly necessary technique for the therapeutic evaluation of small molecules for further processing. Based on this in silico approach, more small molecules have been discovered as potential therapeutic candidates [93]. The increasing failure rate of drug molecules at the clinical trial stage has been attributed to poor ADME/Tox profiles. Hence, predictions of good ADMET profiles of natural compounds can help to eliminate compounds with unacceptable side effects [94]. Therefore, the in silico approach remains the cheapest and most time-efficient model for screening bioactive molecules [95]. In this study, the ADME/Tox and drug-likeness analysis of the phytochemicals from *M. communis* L. was performed to screen out compounds with potential toxicity that violate the drug-likeness rules. Out of the 57 phytochemicals obtained from *M. communis* L. via GC-MS analysis, 27 were predicted to have good pharmacokinetic profiles and obey the Lipinski, Vebers, and Egan rules of drug-likeness.

While several rules have been developed and used to predict the drug-likeness of bioactive compounds from plants, the most prominently used is Lipinski’s rule of five, which was considered in this study. According to this rule, drug-like compounds should not violate more than one of the following rules: a number of H-bond acceptors less than 10, a number of H-bond donors less than 5, a molecular weight less than 500, a partition coefficient less than or equal to 5, and a polar surface area less than or equal to 14 Å [96]. The results from this study (Table 4) reveal that all the hit compounds were positive for human intestinal absorption and Caco-2 and were negative for the P-glycoprotein substrate, CYP2D6 inhibition, and carcinogenicity, while pinostrobin chalcone was the only compound that was negative for blood–brain barrier penetration. Most orally administered drugs exhibit their potency when they are absorbed into the bloodstream for circulation [97]. The ability of bioactive molecules to cross the blood–brain barrier is a significant consideration for the management of neurodegenerative disorders [98]. Human intestinal absorption and Caco-2 remain as the tools for screening the intestinal permeability of therapeutic agents. Hence, the hit compounds in this study can effectively permeate the intestine for metabolism. The membrane P-glycoprotein (P-gp) is known to inhibit the absorption, distribution, and bioavailability of its substrate as small molecules, thereby eliminating them from circulation [93]. The negative result predicted for the hit compounds revealed that they have high bioavailability in the system. The hit compounds were also revealed to be non-carcinogenic, indicating the safety of the plant.

Virtual screening via molecular docking is an in silico model that employs a computer-based tool that has been used in structure-based drug development [93]. It relies on the principle of predicting the binding orientation and evaluating the binding energy of small molecules interacting at the binding pocket of the target. The results of molecular docking presented in Table 4 show better binding affinity of the hit compounds against the inflammatory protein. The hit compounds showed better binding affinity against COX-2 and PLA-2 compared with the standard drug (diclofenac). Hence, these proteins might be predicted as the mechanism of anti-inflammatory action, which corroborates with the in vitro and in vivo anti-inflammatory results obtained in this study.

## 4. Materials and Methods

### 4.1. Chemicals, Reagents, and Equipment

All reagents and solvents used in this study were of analytical grade. The 1,1-diphenyl-2-picrylhydrazyl (DPPH) and 2,2′-azino-bis (3-ethylbenzothiazoline-6-sulfonic acid) (ABTS) free radicals and carrageenan were purchased from Sigma-Aldrich (St. Louis, MO, USA).

### 4.2. Collection of Plant Material

Freshly harvested plant materials (myrtle leaves) were randomly collected in the early morning in April 2022 in Taxana, Jijel, Algeria, and identified as *Myrtus communis* L. The region is located 22 km southeast of Jijel city (latitude: 36° 39′38″; longitude: 5° 47′28″; altitude: 750 m).

### 4.3. Methanolic Extract Preparation

Methanolic extract from leaves of *M. communis* was prepared according to the protocol reported by Dib et al. [99] with slight modifications. Samples were first visually examined for any infection, damage, discoloration, or distortion. Subsequently, to remove all impurities, the plant specimens were subjected to a triple-washing process using tap water followed by distilled water, then shade-dried for 2 weeks at room temperature (37 °C). The dried specimens were weighed and pulverized with a grinding machine. The fine powder samples were preserved in air-tight canisters shielded from light until they were utilized for subsequent procedures. The powder was subjected to extraction by maceration in 96% methanol for 24 h with a ratio of 10:1 (*v*/*w*) of solvent. The extract was then filtered using Wattman filter paper No. 3 to remove plant debris. Samples were centrifuged to obtain a better quality filtrate. A rotary evaporator (BUCHI Rotavapor R-300, BÜCHI, Flawil, Switzerland) was used to remove the methanol from the extract at 45 °C with rotations of 95 to 100 rpm in vacuum. After evaporation, some parts of the extract were stored in an Eppendorf tube at 4 °C for further studies, and the remaining extract was further diluted, filtered, and stored for phytochemical analysis. The extraction yield (EY) is expressed as a percentage of the weight of the obtained dried extract relative to the initial dried matter of the sample used for extraction, as described in the following equation:

$$\%\;\mathrm{EY}=\frac{weight\;of\;dry\;extract}{weight\;taken\;for\;extraction\;}\times 100$$

### 4.4. Gas Chromatography–Mass Spectrometry (CG-MS) Analysis

The phytochemical investigation of the extract was performed using a Shimadzu QP2010 device. For the MMEx sample, the working solution (1 µL) was injected in splitless mode and the temperature program was set as follows: initial temperature at 70 °C for 4 min, then raised and maintained at 240 °C and fitted with a fused-silica SE30 capillary column (length 25 m, internal diameter 0.25 mm, film thickness 0.25 μm). The total run time was 30.25 min. Helium (He) with a purity of 99.99% was selected as an inert carrier gas at a flow rate of 1.4 mL/min. The ion source temperature was set at 200 °C and the interface temperature at 250 °C. The electron impact ionization (EI) was 70 eV and mass spectra were scanned in the range of 40–450 *m*/*z*. Bioactive ingredients in MMEx were identified and authenticated based on the retention time (Rt) with respect to their spectra, then classified by matching the spectral patterns attained with the existing NIST05 2010 mass spectral library. Detected compounds with >90% spectral matching quality were considered acceptable. The determination of the percentage of each component was based on peak area.

### 4.5. Total Polyphenol Content (TPC)

The Folin–Ciocalteu procedure was applied as previously described by Siddiqui et al. [100]. In this procedure, 500 µL of MMEx (1.0 mg/mL) diluted by 1/100th in 2.25 mL of distilled water was treated with 250 µL of 10% Folin–Ciocalteu reagent. After 5 min, 2 mL of 7.5% *w*/*v* sodium carbonate (Na_2_CO_3_) was added. The mixture was vortexed for 15 s before being kept at ambient temperature for 1 h to allow for color development. The interaction between the Folin–Ciocalteu and phenolic residues led to the formation of a complex blue color, with its intensity proportional to the concentration of polyphenols in the extract. Using a spectrophotometer, the extract’s absorbance at 765 nm was detected. The TPC of the extract was calculated using a curve made with gallic acid concentrations ranging from 0 to 400 mg/L; the result is represented as mg of sample gallic acid equivalent (GAE)/g.

### 4.6. Total Flavonoid Content (TFC)

The TFC content was determined using the method outlined by Dewanto et al. [101]. Specifically, 250 μL of the extract (1 mg/mL) was combined with 75 μL of NaNO_2_ (5%). After resting for 6 min at room temperature away from light, 150 μL of AlCl_3_ (2%) and 500 μL of NaOH (1 M) were added, the volume was completed with 2.5 mL of distilled water, and then a spectrophotometer was used to test the mixture’s absorbance at 510 nm. A standard curve with quercetin concentrations ranging from 0 to 200 µg/mL was used to determine TFC, and the results were expressed as milligrams of quercetin equivalent per gram of dry matter (mg QE/g).

### 4.7. Total Hydrolyzable and Condensed Tannin Content (THT, TCT)

HT content was determined according to the protocol of Kardel et al. [102]. For this procedure, 0.2 g of each ground organ was macerated for 18 h in 10 mL of 96% methanol, and the mixture was filtered using Whatman No. 1 paper. Then, 1 mL of the filtrate was added to 3.5 mL of a solution prepared based on 10^−2^ M ferric trichloride (FeCl_3_) in 10^−3^ M hydrochloric acid (HCl). After 15 s, the absorbance of the mixture was read at 660 nm. HT was expressed by the following formula:

$$HT\left(\%\right)=\frac{A.P.M.V.FD}{E\;mole.\;P}$$

where *A* is absorbance; E mole is 2169 for gallic acid (constant expressed in moles); *M* is mass (=300); *V* is the volume of the extract used; *PM* is the molecular weight of gallic acid (170.12 g/mol); *P* is the weight of the sample used; and *FD* is the factor of dilution. The results, represented in the form of percentages (%), were converted into milligram-equivalent gallic acid (EGA) per 100 g of dry matter (mg EGA/100 g MS) [98].

Condensed tannins (CTs) were quantified using the protocol of Martin et al. [103]. For this procedure, 0.2 g of each crushed organ was macerated for 18 h in 10 mL of 96% methanol. The mixture was filtered using Whatman No. 1 paper. Then, 1 mL of filtrate was added to 2 mL of a solution prepared in 1% vanillin base in 70% sulfuric acid. The entire mixture was placed in a water bath for 15 min at 20 °C away from light. The absorbance of the mixture was read at 500 nm. CT content is expressed by the following formula:

$$CT\;\left(\%\right)=5.2\times 10^{-2}\;.\frac{A.V}{P}$$

where 5.2 × 10^−2^ is the cyanidine-equivalent constant, *A* is absorbance, *V* is the volume of extract used, and *P* is the weight of the sample. *CT* is expressed in % or milligram-equivalent cyanidine (EC) per 100 g of dry matter (mg EGA/100 g MS).

### 4.8. Antioxidant Activity

The antioxidant activity of the methanolic extract of *M. communis* was determined by using 5 complementary in vitro assays: DPPH^•^, hydrogen peroxide (H_2_O_2_), hydroxyl radical (OH^•^), 2,2′-azino-bis (3-ethylbenzothiazoline-6-sulfonic acid) (ABTS^•+^), and ferric-reducing antioxidant power (FRAP). This enables a more straightforward and direct comparison of antioxidant activity. Ascorbic acid was used as a reference standard. Six dilutions were performed to obtain concentrations from 0 to 200 µg.mL^−1^. Antioxidant activity was quantified in terms of half-maximal inhibitory concentration (IC_50_), which generally provides outstanding potency information in pharmacological research by indicating how much of the drug is needed to inhibit a biological process by half. A lower IC_50_ value indicates a stronger ability to scavenge free radicals. All measurements were conducted in triplicate (n = 3).

#### 4.8.1. 2,2-Diphenyl-1-picrylhydrazyl (DPPH) Scavenging Assay

The antioxidant potential of MMEx was determined by DPPH free radical-capturing assay using a procedure described by Cosmulescu et al. [104], with some amendments. In brief, 0.5 mL of sample was mixed with 1.5 mL of DPPH-MeOH (0.2 mmol.L^−1^). This was vigorous shaken to obtain a uniform dispersion and kept aside for 30 min in the dark at 37 °C to achieve a steady state of scavenging. The active compounds manifested as yellow marks surrounded by purple. Optical density was recorded at a wavelength of 517 nm. More radical scavenging activity is indicated by a lower degree of absorption. DPPH in methanol solution was used as a control. The inhibition percentage was calculated using Equation (1):

(1) $$I\;\left(\%\right)=\frac{OD_{0}-OD_{1}}{OD_{0}}\times 100$$

where *OD*_0_ is the optical density of the control and *OD*_1_ is the optical density of the sample.

#### 4.8.2. H_2_O_2_ Hydrogen Peroxide Scavenging Assay

This assay was conducted as described by Khan et al. [105]. Briefly, a 40 mM solution of hydrogen peroxide (30%) was prepared in phosphate buffer (PBS) (0.1 M; pH = 7.4). Then, 2 mL of MMEx was added to 1.5 mL of H_2_O_2_ solution. Using a spectrophotometer, the absorbance of the mixture was recorded after 10 min at 230 nm. An extra blank sample without hydrogen peroxide was prepared for background inference. The percentage of H_2_O_2_ inhibition was calculated using Equation (1).

#### 4.8.3. Hydroxyl Radical (OH•) Scavenging Test 

This test was performed according to the typical methodology described by Brands et al. [106] as follows: 1 mL of MMEx was added to the reaction mixture, which consisted of 1 mL of FeSO_4_ (1.5 mM), 0.7 mL of H_2_O_2_ (6 mM), and 0.3 mL of sodium salicylate (20 mM). After 1 h of incubation at 37 °C, the absence of the hydroxylated salicylate complex was measured at 562 nm. The hydroxyl radical scavenging capacity was calculated using Equation (1).

#### 4.8.4. ABTS• Radical Scavenging Assay

According to the protocol described by Dong et al. [107]., the ABTS discoloration technique initially involved converting 7 mM ABTS (colorless) to ABTS^+^ (blue) by adding 2.45 mM K_2_S_2_O_8_. The prepared mixture was left in the dark at room temperature for 12 to 16 h before further use. The resulting solution was diluted in methanol in order to adjust the absorbance at 734 nm to 0.70 ± 0.02 units in ethanol. Then, 1 mL of newly prepared ABTS^•+^ solution was added to 1 mL of sample solution at various concentrations, and the absorbance was recorded 10 min after the initial mixing. Using the same formula as the DPPH test (Equation (1)), the percentage of inhibition was determined.

#### 4.8.5. Ferric-Reducing Antioxidant Power (FRAP)

The ability of MMEx to reduce the ferric iron (Fe^3+^) present in the K_3_Fe (CN)_6_ complex to ferrous iron (Fe^2+^) was tested following the experimental protocol of Suseela et al. [108] with minor changes. For this procedure, 0.5 mL of MMEx or ascorbic acid was mixed with 2.5 mL of PBS (0.2 M; pH 6.6) and 2.5 mL of hexacyanoferrate ([K_3_Fe (CN)_6_]) solution at 1% and incubated at 50 °C for 20 min using a water bath. This step was necessary to reduce ferricyanide into ferrocyanide. Then, 2.5 mL of 10% trichloroacetic acid was added to stop the reaction. The tubes were centrifuged at 3000 rpm for 10 min. Then, 2.5 mL of the upper layer of the mixture was separated and mixed with 2.5 mL of distilled water. Finally, 500 µL of freshly prepared ferric chloride solution (0.1%) was added to the mixture to develop the color. The absorbance at 700 nm was read against a blank after 10 min of incubation at room temperature. The antioxidant activity and IC_50_ were determined.

### 4.9. Anti-Inflammatory Activity

#### 4.9.1. In Vitro: Inhibition of Bovine Serum Albumin (BSA) Denaturation

Inhibition of albumin denaturation was performed to analyze the anti-inflammatory activity of MMEx using a modified version of a method described by Kar et al. [109]. Diclofenac sodium (DCF) was used as a standard. The test consisted of preparing the reaction mixture containing 2.5 mL of PBS (pH = 6.4), 0.5 mL of 5% (*w*/*v*) BSA solution, and 50 μL of the MMEx at different concentrations (200, 100, 50, 25, 12.5, 0 μg.mL^−1^). Samples were taken out after incubation at 37 °C for 15 min and then re-incubated at 70 °C for 5 min to denature the protein. After cooling, the absorbance was read at 600 nm. The following equation was used to determine the percentage of protein denaturation inhibition:

$$I\;\left(\%\right)=\frac{Ac-At}{Ac}\times 100$$

where *Ac* is absorbance of the control and *At* is absorbance of the tested sample. The control represents the 100% denatured protein, and the results were compared with diclofenac sodium.

#### 4.9.2. In Vivo: Carrageenan-Induced Paw Edema in Rats

- *Animals*

The experiment was conducted using female albino Wistar rats (weighing 190 ± 10.0 g) procured from the Pasteur Institute (Algeria). A maximum of 5 rats were housed in each cage, and each animal was given unrestricted access to standard water and pellet food. Every day, the light/dark cycle, temperature, and humidity were maintained (22 ± 2 °C, 55 ± 10%). Before the experiments, rats were acclimatized to laboratory conditions for 7 days.

- *Ethical statement*

The experimental procedure was compliant with internationally accepted protocols and the ethics of laboratory animal care and use as approved by a committee of the Algerian Association of Sciences in Animal Experimentation (No. 8808/1988), and associated with veterinary medical activities and animal-health protection (No. JORA:004/1988).

- *Experimental design*

Before starting, the rats were fasted for 18 h but had free access to water the entire time. According to the protocol of Karim et al. [110] with slight modifications, a single freshly prepared dose of DCF (50 mg.kg^−1^), used as the reference drug, and 2 low doses of MMEx (25–50 mg.kg^−1^) were given to rats orogastrically [29]. Then, 30 min after administration of the appropriate experimental substance, 1% (*w*/*v*) sterile Car dissolved in 0.9% saline solution was administered as a single 0.1 mL injection under the plantar aponeurosis of the right hind footpad of animals in all groups. The basal thickness of the animals was measured just before starting the induction of inflammation (time 0) and then after Car injection at hourly intervals for 4 h, using an electronic digital Vernier caliper. The difference between initial and successive readings represented the rate of inflammation or volume of edema and the percentage of inhibition was measured.

#### 4.9.3. In Silico

- *Ligand Preparation*

The 57 compounds were subjected to drug-likeness screening based on the Lipinski, Ghose, Veber, Egan, and Muegge rules, and the bioavailability of the compounds was determined using the SwissADME website (swissadme.ch). The 3D structures of the 27 compounds and the reference drug diclofenac were downloaded from the NCBI PubChem Compounds database (https://www.pubchem.ncbi.nm.nih.gov/) [111] in SDF format. Using Discovery Studio, the SDF files were saved in PDB format.

- *Protein Preparation*

The target proteins, COX-2 (5F19), IL-1β (4G6M), NF-κB (1NFI), PLA2 (1DCY), and TNF-α (2AZ5), with crystallographic resolutions of 2.04, 1.81, 2.70, 2.70, and 2.10 Å, respectively, were downloaded from the Protein Data Bank (http://rcsb.org) [112]. The target proteins were minimized and constructed using UCSF Chimera software (v 1.16). The binding sites of proteins were predicted using BIOVIA Discovery Studio and published studies [113,114].

- *Molecular Docking Analysis*

Molecular docking analysis was performed and binding affinity was determined using Auto Dock v. 4.2 [115]. The protein targets and ligands in pdbqt format were dragged into their respective columns. All ligands were docked, and a maximum exhaustiveness of 8 was computed for all of them. All other parameters in the software were in default mode. The binding affinity of compounds for the protein targets was recorded. The compounds were then ranked by their docking scores. Also, the molecular interactions between protein targets and compounds were viewed using BIOVIA Discovery Studio. Amino acids engaging with the ligand, hydrogen bonds (H-bonds), hydrophobic interactions, and the individual atoms involved were examined in each ligand cluster (Supplementary Table S2).

### 4.10. In Silico Pharmacokinetic Profile and Drug-Likeness Prediction

The admetSAR web platform (http://lmmd.ecust.edu.cn/admetstar2 (accessed on 29 January 2024)) was used to predict the absorption, distribution, metabolism, and toxicity (ADMET) of the reference drug (DCF) and the hit compounds. The 57 compounds of MMEx were subjected to drug-likeness testing based on the Lipinski, Ghose, Veber, Egan, and Muegge rules and their bioavailability. A total of 30 compounds were screened out, leaving 27 compounds.

## 5. Data Analysis

Data obtained from the laboratory experiment were subjected to one-way analysis of variance (ANOVA) followed by a post hoc LSD test, with a level of significance of *p* < 0.05, using GraphPad Prism 8. All of the results were presented as the mean ± standard deviation.

## 6. Conclusions

After the characterization and quantification of methanolic extract from *Myrtus communis* L., the obtained outputs highlight that myrtle leaves are particularly rich in a plethora of phenolic compounds, with acceptable pharmacokinetics, promising anti-inflammatory activity, substantial antioxidant potential, and excellent safety, paving the way for an exciting new chemical entity. According to these data, we believe that the investigated MMEx could be considered to be an alternative agent for the treatment of chronic inflammation instead of NSAIDs, as their anti-inflammatory potency is comparable, but they do not produce the acute side effects of DCF. It would also be interesting to test a larger range of extract concentrations to determine the lowest concentration that would maintain the anti-inflammatory effects. For this purpose, well-designed animal and clinical studies are needed to verify the clinical benefits of this plant extract over the long term. It is necessary to guarantee the quantifiable and uniform content of active substances in phyto-medicines to ensure the desired pharmacological effect and the pharmacokinetic behavior, and to establish doses and therapeutic regimens with reduced adverse effects.

## Figures and Tables

**Table 1:** Contents of total polyphenols, flavonoids, and tannins in *Myrtus communis* L. methanolic extract.

Sample	Polyphenols (mg EAG.g^−1^)	Flavonoids (mg QUE. g^−1^)	Condensed Tannins (CT) (%)	Hydrolysable Tannins (HT) (%)
**MMEx**	415.85 ± 15.52	285.80 ± 1.64	0.90	0.68

**Table 2:** Chemical composition of MMEx by GC–MS. Rt, retention time.

Peak	Compound	Chemical Formula	(%)	Rt	*m*/*z*
**1** **2** **3** **4** **5** **6** **7** **8** **9** **10** **11** **12** **13** **14** **15** **16** **17** **18** **19** **20** **21** **22** **23** **24** **25** **26** **27** **28** **29** **30** **31** **32** **33** **34** **35** **36** **37** **38** **39** **40** **41** **42** **43** **44** **45** **46** **47** **49** **50** **51** **52** **53** **54** **55** **56** **57**	Alpha-pinene Isobutyl isobutyrate D-limonene Iso-butyl-2-methylbutyrate 1,8-Cineole 2-Methylbutyl 2-methylbutyrate Linalool Trans-pinocarveol Phenylethyl alcohol (-)-Terpinen-4-ol Estragole Terpneol Linalyl acetate (-)-Cis-carveol (+-)-Pulegone Geraniol Terpinyl acetate Geranyl acetate Caryophyllene Ethanone, 1-(2-hydroxy-5-methylphenyl)- Coumaran Alpha-caryophyllene Chavibetol Methyl eugenol (Hydroxymethyl)ethylene acetate Cyclohexanecarboxaldehyde, 6-methyl-3-(1-methylethyl)-2-xo-1-(3-oxobutyl)- 2,4-Hexanedione, 5-methyl-3-(2-methyl-1-ropenyl)- Durohydroquinone O-eugenol Caryophyllene oxide Cedrol Hedycaryol 1, 2,4-Cyclopentanetrione, 3-(2-pentenyl)- Benzaldehyde, 2-hydroxy-4-methyl- 2-Pentadecanone, 6, 10,14-trimethyl-(or phytone) 2-Naphthalenecarboxylic acid, 3,4-dihydro- β-selinenol 2-O-tosyl-1,3,4,6-tetra-o-acetyl-alpha-d-galactose Isobutyl phthalate Isopropyl palmitate Palmitic acid Dibutyl phthalate 1-Naphthalene propanol alp Cadalene p-Dimethylaminobenzophenone Elaidic acid, isopropyl ester Isopropyl stearate Tetracosane Oleic acid Sulfuric acid, octadecyl 2-propyl ester Eicosane Thunbergol Totarol Cinnamyl cinnamate Pinostrobin chalcone Tetratriacontane Total	C_10_H_16_ C_8_H_16_O_2_ C_10_H_16_ C_9_H_18_O_2_ C_10_H_18_O C_10_H_20_O_2_ C_10_H_18_O C_10_H_16_O C_8_H_10_O C_10_H_18_O C_10_H_12_O C_10_H_18_O C_12_H_20_O_2_ C_10_H_16_O C_10_H_16_O C_10_H_18_O C_12_H_20_O_2_ C_12_H_20_O_2_ C_15_H_24_ C_12_H_20_O_2_ C_9_H_10_O_2_ C_8_H_8_O C_15_H_24_ C_10_H_12_O_2_ C_11_H_14_O_2_ C_7_H_12_O_2_ C_15_H_24_O_3_ C_11_H_18_O_2_ C_10_H_14_O_2_ C_10_H_12_O_2_ C_15_H_24_O C_15_H_26_O C_15_H_26_O C_10_H_12_O_3_ C_8_H_8_O_2_ C_18_H_36_O C_18_H_36_O C_15_H_26_O C_21_H_26_O_12_S C_16_H_22_O_4_ C_19_H_38_O_2_ C_16_H_32_O_2_ C_16_H_22_O_4_ C_20_H_34_O C_15_H_18_ C_15_H_15_NO C_21_H_40_O_2_ C_21_H_42_O_2_ C_24_H_50_ C_20_H_42_ C_21_H_44_O_3_S C_18_H_34_O_2_ C_20_H_34_O C_20_H_30_O C_18_H_16_O_2_ C_16_H_14_O_4_ C_34_H_70_ -	10.06 0.23 0.52 0.23 33.8 0.26 4.83 0.18 0.31 0.16 0.19 3.60 0.85 0.13 0.16 1.00 0.64 0.16 0.49 3.25 0.14 0.19 0.25 2.48 1.25 0.17 0.19 0.12 0.67 0.55 0.14 0.21 0.41 0.30 0.27 0.45 0.25 0.36 0.28 0.34 2.44 2.97 0.17 0.73 0.16 4.21 2.01 0.19 0.58 1.78 1.26 1.70 4.00 3.30 0.23 1.80 2.42 99.72	3.079 3.477 5.365 5.529 5.883 7.920 8.687 9.585 10.073 10.310 10.465 10.724 11.234 11.543 11.663 12.053 12.976 13.247 13.505 13.595 13.848 13.970 14.144 14.258 14.649 15.334 15.985 16.626 16.971 17.100 17.222 17.370 18.225 18.475 18.697 20.321 21.152 21.394 21.492 21.590 22.032 22.656 22.793 23.182 23.305 23.680 24.082 24.256 24.321 24.714 25.355 26.500 26.647 26.850 28.726 29.347 29.597 -	93.05 71.05 68.05 57.05 43.00 85.05 71.05 92.05 71.05 91.05 148.15 59.05 93.10 109.10 81.05 69.10 121.10 43.00 93.10 69.10 150.10 120.05 93.10 164.10 178.10 43.00 139.10 43.05 166.10 164.10 43.00 95.10 59.05 180.10 136.05 43.00 129.10 149.10 91.10 149.05 43.05 73.05 149.05 81.05 183.10 326.15 55.00 102.05 57.10 55.05 57.05 57.05 81.10 271.20 131.05 270.10 57.10 -

**Table 3:** Docking scores of compounds and standard drugs within binding pocket of pro-inflammatory receptors.

Ligand	Docking Scores (kcal/mol)				
	COX-2	IL-1β	NF-κB	PLA2	TNF-α
**Diclofenac**	−7.7	−6.1	−6.1	−7.7	−5.7
**Pinostrobin chalcone**	−8.8	−5.7	−5.7	−7.4	−6.2
**Cinnamyl cinnamate**	−9.5	−5.5	−6.5	−8.0	−6.1
**Hedycaryol**	−7.3	−6.2	−5.8	−7.2	−6.2
**Totarol**	−8.0	−6.2	−7.1	−8.1	−7.0
**P-dimethylaminobenzophenone**	−8.5	−6.1	−6.4	−8.5	−6.5

**Table 4:** ADMET profiling of hit compounds in relation to diclofenac according to admetSAR.

ADMET Properties	Diclofenac	Cinnamyl Cinnamate	Pinostrobin Chalcone	Hedycaryol	Totarol	P-Dimethyl- Aminobenzophenone
**Ames mutagenesis**	-	+	-	+	-	-
**Acute oral toxicity (c)**	II	III	III	IV	III	III
**Blood–brain barrier**	+	+	-	+	+	+
**Caco-2**	+	+	+	+	+	+
**Carcinogenicity**	-	-	-	-	-	-
**CYP1A2 inhibition**	+	+	+	-	+	-
**CYP2C19 inhibition**	-	+	+	-	-	-
**CYP2C9 inhibition**	+	-	+	-	-	-
**CYP2C9 substrate**	+	-	-	-	+	-
**CYP2D6 inhibition**	-	-	-	-	-	-
**CYP2D6 substrate**	-	-	-	-	+	-
**CYP3A4 inhibition**	-	-	+	-	-	-
**CYP3A4 substrate**	-	-	-	-	+	+
**CYP inhibitory promiscuity**	-	+	+	-	-	-
**Hepatotoxicity**	+	-	+	+	-	+
**Human ether-a-go-go-related gene inhibition**	-	-	-	+	-	+
**Human intestinal absorption**	+	+	+	+	+	+
**Human oral bioavailability**	+	-	-	+	-	-
**Nephrotoxicity**	+	-	-	-	-	-
**Acute oral toxicity**	2.782	1.575	2.181	1.198	3.08	2.096
**P-glycoprotein inhibitor**	-	-	-	-	-	+
**P-glycoprotein substrate**	-	-	-	-	-	-
**Plasma protein binding**	1.01 (101%)	0.767 (76.7%)	0.973 (97.3%)	0.852 (85.2%)	0.955 (95.5%)	0.779 (77.9%)
**Subcellular localization**	Mitochondria	Mitochondria	Mitochondria	Lysosomes	Mitochondria	Mitochondria
**UGT catalysis**	-	-	+	-	-	-
**Water solubility**	−4.467	−3.823	−3.1	−2.976	−4.28	−2.652

## Data Availability

Data are contained within the article and Supplementary Materials.

## Supplementary Materials

The following supporting information can be downloaded at: https://www.mdpi.com/article/10.3390/molecules29040849/s1. Table S1. Druglikeness of Compounds from plants using SwissAdme Server. Table S2. The grid center, dimensions of each target proteins and the amino acid residue present at the active site.
