# Supplementary material for: Chemical Composition Antioxidant and Anti-Inflammatory Activities of Myrtus communis L. Leaf Extract: Forecasting ADMET Profiling and Anti-Inflammatory Targets Using Molecular Docking Tools
Source: Molecules. 2024 Feb 14;29(4):849. doi: 10.3390/molecules29040849 (PMC10893115; doi:10.3390/molecules29040849)
Supplement: Supplementary file 1 [file molecules-29-00849-s001.zip › molecules-2765635-supplementary.pdf]

Supplementary Informations (SI)

Table S1. Druglikeness of Compounds from plants using SwissAdme Server.

S/N	Compounds	MW	Lipinski Violations	Veber Violations	Egan Violations	Muegge Violations	Ghose Violations	Bioavailability
**1**	Alpha-Pinene	136.23	1	0	0	2	1	0.55
**2**	Isobutyl Isobutyrate	144.21	0	0	0	1	1	0.55
**3**	D-Limonene	136.23	0	0	0	2	1	0.55
**4**	Iso-Butyl-2-Methylbutyrate	158.24	0	0	0	1	1	0.55
**5**	1,8-Cineole	154.25	0	0	0	1	2	0.55
**6**	2-Methylbutyl 2-Methylbutyrate	172.26	0	0	0	1	0	0.55
**7**	Linalool	154.25	0	0	0	2	1	0.55
**8**	Trans-Pinocarveol	152.23	0	0	0	2	1	0.55
**9**	(-)-Terpinen-4-Ol	154.25	0	0	0	2	1	0.55
**10**	Phenylethyl Alcohol	122.16	0	0	0	2	3	0.55
**11**	Estragole	148.20	0	0	0	2	1	0.55
**12**	Terpineol	154.25	0	0	0	2	1	0.55
**13**	Linalyl Acetate	196.29	0	0	0	1	0	0.55
**14**	(-)-Cis-Carveol	152.23	0	0	0	2	1	0.55
**15**	(+-)-Pulegone	152.23	0	0	0	2	1	0.55
**16**	Geraniol	154.25	0	0	0	2	1	0.55
**17**	Terpinyl Acetate	196.29	0	0	0	1	0	0.55
**18**	Geranyl Acetate	196.29	0	0	0	1	0	0.55
**19**	Caryophyllene	204.35	1	0	0	1	0	0.55
**20**	Ethanone, 1-(2-Hydroxy-5-Methylphenyl)-	150.17	0	0	0	1	1	0.55
**21**	Coumaran	120.15	0	0	0	2	3	0.55
**22**	Alpha.-Caryophyllene	222.37	0	0	0	1	0	0.55
**23**	Chavibetol	164.20	0	0	0	1	0	0.55
**24**	Methyl Eugenol	178.23	0	0	0	1	0	0.55
**25**	(Hydroxymethyl)Ethylene Acetate	176.17	0	0	0	1	2	0.55
**26**	Cyclohexanecarboxaldehyde, 6-Methyl-3-(1-Methylethyl)-2-Oxo-1-(3-Oxobutyl)-	252.35	0	0	0	0	0	0.55
**27**	2,4-Hexanedione, 5-Methyl-3-(2-Methyl-1-Propenyl)-	182.26	0	0	0	1	0	0.55
**28**	Durohydroquinone	166.22	0	0	0	1	0	0.55
**29**	O-Eugenol	164.20	0	0	0	1	0	0.55
**30**	Caryophyllene Oxide	220.35	0	0	0	1	0	0.55
**31**	Cedrol	227.35	0	0	0	1	0	0.55
**32**	Hedycaryol	227.35	0	0	0	1	0	0.55
**33**	1, 2,4-Cyclopentanetrione, 3-(2-Pentenyl)-	180.20	0	0	0	1	0	0.55
**34**	Benzaldehyde, 2-Hydroxy-4-Methyl-	136.15	0	0	0	1	3	0.55
**35**	2-Pentadecanone, 6, 10,14-Trimethyl- (Or Phytone)	268.5	1	1	1	2	1	0.55
**36**	2-Naphthalenecarboxylic Acid, 3,4-Dihydro-	174.20	0	0	0	1	0	0.55
**37**	β-Selinenol	227.37	0	0	0	1	0	0.55
**38**	2-O-Tosyl-1,3,4,6-Tetra-O-Acetyl-.Alpha.-D-Galactose	502.5	1	2	1	2	1	0.17
**39**	Isobutyl Phthalate	278.34	0	0	0	0	0	0.55
**40**	Isopropyl Palmitate	298.50	1	1	1	2	1	0.55

**41**	Palmitic Acid	256.42	1	1	0	1	0	0.85
**42**	Dibutyl Phthalate	278.34	0	0	0	0	0	0.55
**43**	1-Naphthalene propanol,alp	290.5	1	0	0	2	0	0.55
**44**	Cadalene	198.30	1	0	0	3	0	0.55
**45**	P-Dimethylaminobenzophenone	326.4	0	0	0	0	0	0.55
**47**	Elaidic Acid, Isopropyl Ester	324.5	1	1	1	2	1	0.55
**48**	Isopropyl Stearate	326.6	1	1	1	2	1	0.55
**49**	Tetracosane	338.7	1	1	1	3	2	0.55
**50**	Oleic Acid	282.5	1	1	1	1	1	0.85
**51**	Sulfurous Acid, Octadecyl 2-Propyl Ester	376.6	1	1	1	2	1	0.55
**52**	Eicosane	282.5	1	1	1	3	1	0.55
**53**	Thunbergol	290.5	1	0	0	2	1	0.55
**54**	Totarol	286.5	1	0	0	2	0	0.55
**55**	Cinnamyl Cinnamate	264.3	0	0	0	0	0	0.55
**56**	Pinostrobin Chalcone	270.68	0	0	0	0	0	0.55
**57**	Tetratriacontane	478.9	1	1	1	3	3	0.55

Table S2. The grid center, dimensions of each target proteins and the amino acid residue present at the active site.

**Protein targets**	**COX-2**	**IL-1β**	**NF-KB**	**PLA2**	**TNF-α**
**Grid centers**	x = 24.7551<br>y= 29.0800<br>z = 65.1509	x = 26.8543<br>y = 29.5456<br>z = 30.0129	x = -2.7435<br>y = 47.3164<br>z = -20.4619	x = 57.8597<br>y = 30.6213<br>z = 41.3366	x = -21.0379<br>y = 69.9000<br>z = 37.4619
**Dimension of the grid box**	x = 2.5571<br>y = 43.3537<br>z = -33.8166	x = 34.0786<br>y = 22.1559<br>z = 31.9965	x = 25.0<br>y = 25.0<br>z = 50.9518	x = 29.2744<br>y = 30.6970<br>z = 25.0	x= 23.3619<br>y = 20.599<br>z = 18.9137
**Amino acid at the active sites**	Thr 212, Phe310, His386, His388, Ala 202, Trp 387, Leu 390, Val295, Phe 200, Ile 408, Gln 203.	Asp 35, Gln 34, Glu 37, Gln 38, Lys65, Asn12 Lys 27, Glu 64 Asn66, Asp 86.	Lys301, Arg304, Arg 302, Phe309, Thr305, Ile-312, Met313, Phe318.	Lys62, His47, Tyr51, Gly29, His27, Phe5, Leu2, Cys44, Cys28, Ala17, Phe98, Ala18.	Gly121, Leu57, Tyr 59, Ser60, Gln61, Tyr119, Leu120, Gly122, Tyr151.
